# Acute Paraxanthine Ingestion Improves Cognition and Short-Term Memory and Helps Sustain Attention in a Double-Blind, Placebo-Controlled, Crossover Trial

**DOI:** 10.3390/nu13113980

**Published:** 2021-11-09

**Authors:** Choongsung Yoo, Dante Xing, Drew Gonzalez, Victoria Jenkins, Kay Nottingham, Broderick Dickerson, Megan Leonard, Joungbo Ko, Mark Faries, Wesley Kephart, Martin Purpura, Ralf Jäger, Shawn D. Wells, Ryan Sowinski, Christopher J. Rasmussen, Richard B. Kreider

**Affiliations:** 1 Human Clinical Research Facility, Exercise & Sport Nutrition Lab, Department of Health & Kinesiology, Texas A&M University, College Station, TX 77843, USA; choongsungyoo@tamu.edu (C.Y.); dantexing@tamu.edu (D.X.); dg18@tamu.edu (D.G.); victoria.jenkins@tamu.edu (V.J.); kvnottingham@tamu.edu (K.N.); dickersobl5@email.tamu.edu (B.D.); meganleonard10@tamu.edu (M.L.); joungboko10@tamu.edu (J.K.); Mark.Faries@ag.tamu.edu (M.F.); ryansowinski6@gmail.com (R.S.); crasmussen@tamu.edu (C.J.R.); 2 Texas A&M AgriLife Extension, Texas A&M University, College Station, TX 77843, USA; 3 Department of Kinesiology, University of Wisconsin-Whitewater, Whitewater, WI 53190, USA; kephartw@uww.edu; 4 Increnovo LLC, Milwaukee, WI 53202, USA; martin.purpura@increnovo.com (M.P.); ralf.jaeger@increnovo.com (R.J.); shawn@ingeniousingredients.com (S.D.W.); 5 Ingenious Ingredients L.P., Lewisville, TX 75056, USA

**Keywords:** mental performance, nootropics, ergogenic aid, caffeine alternative

## Abstract

This study examined the effects of acute paraxanthine (PXN) ingestion on markers of cognition, executive function, and psychomotor vigilance. In a randomized, double blind, placebo-controlled, crossover, and counterbalanced manner, 13 healthy male and female participants were randomly assigned to consume a placebo (PLA) or 200 mg of PXN (ENFINITY™, Ingenious Ingredients, L.P.). Participants completed stimulant sensitivity and side effect questionnaires and then performed the Berg Wisconsin Card Sorting Test (BCST), the Go/No-Go test (GNG), the Sternberg task test (STT), and the psychomotor vigilance task test (PVTT). Participants then ingested one capsule of PLA or PXN treatment. Participants completed side effect and cognitive function tests after 1, 2, 3, 4, 5, and 6 h after ingestion of the supplement. After 7 days, participants repeated the experiment while consuming the alternative treatment. Data were analyzed by general linear model (GLM) univariate analyses with repeated measures using body mass as a covariate, and by assessing mean and percent changes from baseline with 95% confidence intervals (CIs) expressed as means (LL, UL). PXN decreased BCST errors (PXN −4.7 [−0.2, −9.20], *p* = 0.04; PXN −17.5% [−36.1, 1.0], *p* = 0.06) and perseverative errors (PXN −2.2 [−4.2, −0.2], *p* = 0.03; PXN −32.8% [−64.4, 1.2], *p* = 0.04) at hour 6. GNG analysis revealed some evidence that PXN ingestion better maintained mean accuracy over time and Condition R Round 2 response time (e.g., PXN −25.1 [−52.2, 1.9] ms, *p* = 0.07 faster than PLA at 1 h), suggesting better sustained attention. PXN ingestion improved STT two-letter length absent and present reaction times over time as well as improving six-letter length absent reaction time after 2 h (PXN −86.5 ms [−165, −7.2], *p* = 0.03; PXN −9.0% [−18.1, 0.2], *p* = 0.05), suggesting that PXN enhanced the ability to store and retrieve random information of increasing complexity from short-term memory. A moderate treatment x time effect size (η_p_^2^ = 0.08) was observed in PVTT, where PXN sustained vigilance during Trial 2 after 2 h (PXN 840 ms [103, 1576], *p* = 0.03) and 4 h (PXN 1466 ms [579, 2353], *p* = 0.002) compared to PL. As testing progressed, the response time improved during the 20 trials and over the course of the 6 h experiment in the PXN treatment, whereas it significantly increased in the PL group. The results suggest that acute PXN ingestion (200 mg) may affect some measures of short-term memory, reasoning, and response time to cognitive challenges and help sustain attention.

## 1. Introduction

Upon ingestion, caffeine (CA) is metabolized primarily by demethylation to dimethylxanthines. Paraxanthine (1,7-dimethylxanthine, PXN) accounts for 70–72% of CA ingested, and 85% of the methylxanthine metabolic by-products [1], with smaller percentages of CA being metabolized to theobromine (3,7-dimethylxanthine, TB) and theophylline (1,3-dimethylxanthine, TP). PXN is a natural dietary component that can be found in Theobroma cacao fruits [2], Coffea arabica [3], Sinomenium actum [4], and the stamens of citrus flowers [5]. The pharmacokinetics of TB and TP are similar and significantly different from CA and PXN, with PXN having the shortest half-life and greatest plasma clearance [6]. PXN has lower toxicity [7] and lesser anxiogenic effects than CA [8]. Other caffeine metabolites such as TP often induce nausea, diarrhea, tachycardia, and arrhythmias [9].

CA induces locomotor activation by its ability to block adenosine receptors, and, as with CA, PXN is a central nervous stimulant; however, it has higher binding potencies for adenosine A1 and A2a receptors [10]. Consequently, PXN has a stronger locomotor-activating effect than CA, TB, and TP [11]. PXN has been shown to protect dopaminergic neurons and is strongly protective against neurodegeneration and the loss of synaptic function, whereas CA only provides marginal protection [12]. The wake-promoting potency of PXN was greater and longer lasting than that of an equimolar concentration of CA in a mouse model of narcolepsy, and higher doses of CA induced hypothermia and reduced locomotor activity, while PXN did not [13]. By eliminating metabolism into TB and TP, PXN is more than just safer than CA; preclinical studies indicate that PXN might also be more effective than CA.

Currently, only two studies in humans have been performed with PXN ingestion [6,8], investigating PXN’s pharmacokinetics [6] and sympathomimetic properties [8], while studies investigating the potential effects on cognition are currently lacking. The purpose of this study was to examine the effects of acute PXN ingestion (200 mg) on markers of cognition, executive function, and psychomotor vigilance. The rationale for this dosage was that previous reports indicated that the effective dose of CA is about 3–6 mg/kg (i.e., 210–420 mg for a 70 kg individual) [14]. Therefore, a 200 mg dose of PXN would be equivalent to consuming about 285 mg of CA. We hypothesized that since about 70% of CA is metabolized to PXN and it possess some sympathomimetic properties, acute PXN ingestion would promote significant improvements in markers of cognition, memory, and vigilance.

## 2. Methods

### 2.1. Experimental Design

This study was conducted in a university setting as a double-blind, placebo-controlled, crossover trial. This study was conducted with approval from the university’s institutional review board (IRB2019-0453D) in compliance with the Declaration of Helsinki standards for ethical principles regarding human participant research. This study was also registered with the ISRCTN registry (#ISRCTN66975000). Nutritional supplementation served as the independent variable. Primary outcomes included assessment of cognitive and executive function. Secondary outcomes included side effect and adverse event assessments.

### 2.2. Participants

**Figure 1:**
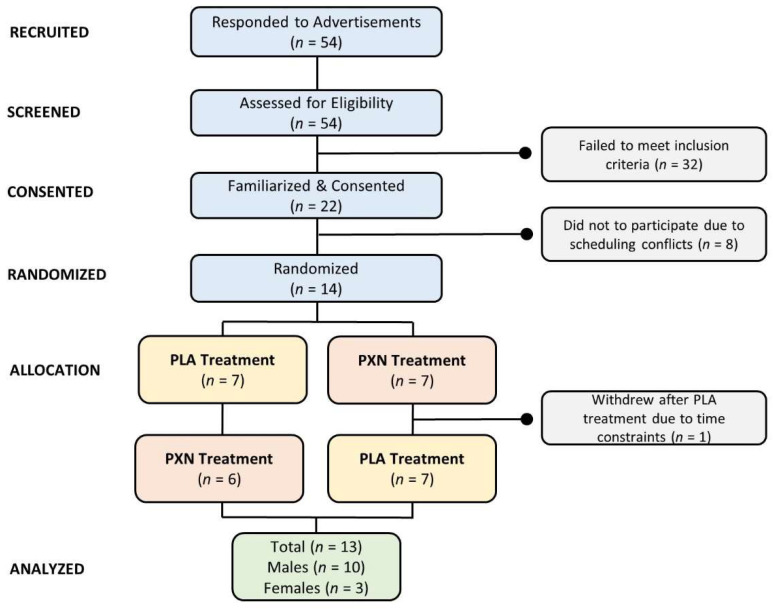
Consolidated Standards of Reporting Trials (CONSORT) diagram of the study.

Healthy males and females between the ages of 18 and 59 were recruited to participate in the study. All participants were healthy and free from known (1) cognitive deficit conditions; (2) wheat flour allergies; (3) sleep disorders; (4) cardiovascular, metabolic, or pulmonary diseases; (5) history of hypertension, migraine headaches, cardiac arrhythmias, or anxiety; and (6) gastrointestinal reflux disease or ulcers. Participants who were taking prescription medications in the month prior to the initiation of the study and/or were told by a physician to abstain or restrict caffeine and/or stimulant intake were excluded from the present study. A minimal sample size of 10 was determined based on the expectation of a 5% improvement in cognitive function markers with a corresponding power of 0.80 based on similar studies assessing nootropics on cognitive and executive function [15,16,17,18,19,20,21,22]. Participants were recruited from the university and local community via direct email and newspaper advertisements as well as by posting study flyers. Volunteers who expressed interest in participating in the study underwent a phone screening to determine general eligibility. Those meeting phone screening entry criteria were invited to participate in a familiarization session. Figure 1 presents a Consolidated Standards of Reporting Trials (CONSORT) diagram of the study. A total of 54 individuals responded to study advertisements and underwent phone screening to assess eligibility. Of these, 22 individuals were familiarized with and consented to participate in the study. A balanced Latin square generator program was used by one of the investigators to randomize participants to treatments in a repeated measures, counterbalanced, and crossover manner [23]. Eight individuals had time constraints and were unable to be scheduled for testing. A total of 14 participants were randomized and allocated into treatments (7 in PLA and 7 in PXN). One participant withdrew after completing the first experiment in the PLA treatment due to time constraints. A total of 13 participants completed the study (10 males and 3 females). Participants were healthy males and females (24 ± 5 years, 170.0 ± 12 cm, 72.9 ± 19 kg, 24.8 ± 4 kg/m^2^).

### 2.3. Testing Protocol

**Figure 2:**
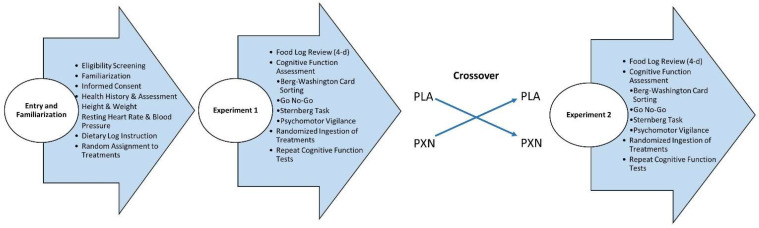
Overview of study protocol.

Figure 2 provides an overview of the experiment protocol. Participants meeting phone screening criteria participated in a familiarization session in which they were informed about the study protocol, signed informed consent statements, and underwent general health screening that included measurement of height, weight, heart rate, and blood pressure. This included having participants complete a comprehensive medical history and report any health issues including any sleep disorders. They were then instructed on how to record food and fluid intake on record forms. Additionally, participants were provided a list of caffeine-containing beverages and common foods to avoid prior to each experiment. Participants then practiced each cognitive function test three times to familiarize them with each test used to assess cognitive and executive function and determine test–retest reliability. Prior to each testing session, participants recorded food and fluid intake for 4 days so they could replicate food and fluid intake prior to each testing session. They were also asked to refrain from consuming atypical amounts of caffeine (i.e., >200 mg/d) and other stimulants not normally consumed in their diet for 48 h and fast for 8 h prior to each experimental session. During each testing session, participants performed baseline cognitive function tests, ingested the assigned treatment, and then repeated the cognitive function tests after 1, 2, 3, 4, 5, and 6 h. Participants were also asked to report any unusual symptoms or side effects. Participants then observed a 4- to 7-day washout period while replicating their 4-day diet with the dietary restrictions noted above, before repeating the experiment with the alternate supplement treatment.

### 2.4. Supplementation Protocol

Supplements were administered in a double-blind, crossover, and randomized manner. Participants ingested capsules containing 200 mg of a wheat flour placebo (PLA, Shandong Bailong Chuangyuan Bio-tec Co. Ltd., Dezhou, China) or 200 mg of paraxanthine (PXN, ENFINITY™, Ingenious Ingredients, L.P Lewisville, TX, USA). Supplements were packaged in similar sized and colored capsules and placed in generically labeled bottles for double-blind administration. Participants observed a 4- to 7-day washout period between experimental sessions and then repeated the experiment while consuming the alternate treatment.

## 3. Procedures

### 3.1. Demographics

Height and weight were measured on a Health-O-Meter Professional 500 KL (Pelstar LLC, Alsip, IL, USA) self-calibrating digital scale (±0.02 kg). Resting hemodynamic measures were obtained in a seated position following approximately 6 min of rest. Heart rate was assessed via the radial artery, while blood pressure was measured by oscillation of the brachial artery using a mercurial sphygmomanometer following standard procedures [24].

### 3.2. Dietary Assessment

Diet intake was assessed via a self-recorded account of all food and energy-containing beverages over a 4-day period prior to the first testing session using the 2021 MyFitnessPal Calorie Counter smartphone app (MyFitnessPal, Inc., Baltimore, MD, USA) or written food logs. The 4-day diet was used to replicate food and beverage intake prior to the next experimental session. Food records were entered by study researchers, verified for consistency by one individual, and analyzed using the Food Processor Nutrition Analysis Software, Version 11.4.412 (ESHA Nutrition Research, Salem, OR, USA) [25].

### 3.3. PEBL Cognitive and Executive Function Assessment

**Figure 3:**
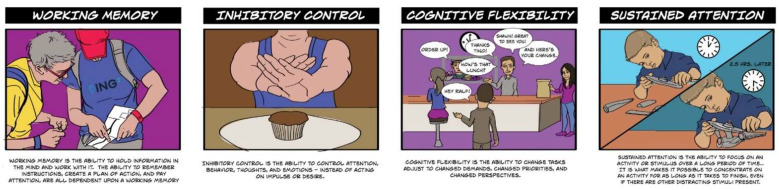
Executive function tests and how they relate to daily activities. (Illustration by Stephen Somers, Milwaukee, WI, USA).

The Psychology Experiment Building Language (PEBL) software program (Version 2.1, Available online: http://pebl.sourceforge.net, accessed on 7 April 2019) was used to administer the cognitive function test battery. The PEBL test comprised four cognitive function tests that assessed a range of cognitive and executive function aspects. The first test administered was the Berg Wisconsin Card Sorting Test (BCST). In this test, participants are presented visual stimuli (i.e., pictures of playing cards) with instructions to sort the cards by matching colors and/or designs [26,27]. The test assesses reaction time and accuracy in measuring reasoning, learning, executive control, attention shifting by assessing inability to shift set (i.e., display flexibility in the face of changing schedules of reinforcement), and impulsiveness [28,29]. Test–retest reliability established during familiarization testing revealed a coefficient of variation (C_V_) of 4.7%, and a standard error of the mean expressed as a percent of the mean (SEM%) of 0.6% for correct responses. The Go/No-Go test (GNG) was then administered. This test assesses sustained attention and response control through reaction time and accuracy of responding to visual stimuli (i.e., seeing P or R) by either pressing a key representing “Go”, or inhibiting a response by not pressing the key representing “No-Go” [26,27,30]. Test–retest reliability of mean accuracy revealed a C_V_ of 6.1% and an SEM% of 0.72. Following this, participants took the Sternberg task test (STT). Visual stimuli are presented one at a time with the participant identifying them as either present or absent within sequences of 3, 6, 9, 12, 15, or 18 s intervals. In order to prevent rehearsal, the participants were instructed to count backwards in threes and fours to a specific random number until they saw a red light appear on the computer screen [26,27]. This test measures short-term/working memory involving cognitive control processes, using reaction time and accuracy [31]. Test–retest reliability of present accuracy during the familiarization session revealed C_V_s of 2.4%, 2.2%, and 4.2% and an SEM% of 0.28, 0.27, and 0.39 for 2-letter, 4-letter, and 6-letter responses, respectively. Participants then performed the general attention psychomotor vigilance task test (PVTT). This test assesses sustained attention reaction times through responses to visual stimuli (as light), requiring participants to press a keyboard button in response to a randomly illuminating light on screen every few seconds [32,33,34]. The number of times the button was not pressed and the speed of the response were measured, with sleepiness quantified as the number of lapses in attention during the test [26,27]. Test–retest reliability during the familiarization session for trials 2, 10, and 20 revealed C_V_s of 41.9%, 36.9%, and 41.6% and an SEM% of 5.01, 4.41, and 4.97, respectively. Figure 3 provides an illustration of how executive function tests relate to daily activities.

### 3.4. Adverse Event Monitoring

Participants were asked to report any unusual symptoms or side effects experienced upon completing each experimental testing session.

### 3.5. Statistical Analysis

Data were analyzed using IBM^®^ SPSS^®^ Version 28 software (IBM Corp., Armonk, NY, USA). Data are reported without sex as a dependent variable since participants served as their own control and there were no significant treatment × time × sex interactions observed. Missing raw data (0.01%) were extrapolated from the average of one time point immediately before and after, wherever possible. Data were analyzed using general linear models (GLMs) with repeated measures univariate and multivariate analyses using body weight (kg) as a covariate. Delta (∆) change values from baseline were calculated and used to determine changes from baseline. Multivariate and univariate effects are expressed through Wilks’ lambda distributions and Greenhouse–Geisser correction tests, respectively, for time (T) and treatment × time (G × T) effects. Data were considered statistically significant when the probability of type I error (α-level) was 0.05 or less, with trends being noted when p-levels ranged between *p* > 0.05 and *p* < 0.10. Fisher’s least significant difference post hoc analysis was performed for pairwise comparisons. Additionally, mean changes from baseline as well as mean percent changes from baseline with 95% confidence intervals (CIs) and Sidak adjustment were calculated. Mean changes and 95% CIs completely above or below baseline were considered significantly different [35,36]. Data are presented as mean or mean change ± SD as appropriate, with figures showing 95% CIs (mean change ± SD [LL, UL]). Partial eta-squared effect sizes ($\eta_{p}^{2}$) are reported as indicators of the magnitude of effect, where 0.01 is considered a small effect, 0.06 is considered a medium effect, and 0.14 is considered a large effect size [37].

## 4. Results

### 4.1. Demographic Data

Table S1 presents participant demographic data. A total of 13 individuals completed the study (3 females and 10 males). Participants were 23.6 ± 5 years old, 170 ± 12 cm tall, weighed 72.9 ± 18.9 kg, had a body mass index (BMI) of 24.8 ± 3.6 kg/m^2^, and had a resting heart rate of 73.2 ± 9 bpm, a systolic blood pressure of 110.8 ± 14 mmHg, and a diastolic blood pressure of 68.2 ± 9 mmHg. Significant sex differences were observed in age (*p* = 0.027), height (*p* < 0.001), weight (*p* < 0.001), BMI (*p* = 0.01), systolic blood pressure (*p* < 0.001), and diastolic blood pressure (*p* = 0.010). For this reason, body weight was used as a covariate in GLM analyses.

### 4.2. PEBL Cognitive Function Assessment

#### 4.2.1. Berg Wisconsin Card Sorting Test

Table S2 presents the results of the BCST that assesses thought, reasoning, learning, executive control, and attention shifting. No significant overall or univariate treatment × time interaction effects were observed from GLM analysis using weight as a covariate in correct responses, errors, perseverative errors (PEBL), or perseverative errors (PAR rules). However, there was evidence that the number of errors decreased over time with PXN treatment, and PAR rule errors were lower with PXN treatment after 6 h (PLA 10.38 ± 3.2, PXN 8.17 ± 1.5, *p* = 0.034). Analysis of mean changes from baseline revealed that errors with PXN treatment were lower after 6 h (−4.76 [−0.2, −9.2], *p*= 0.041).

#### 4.2.2. Go/No-Go Task Test

**Figure 4:**
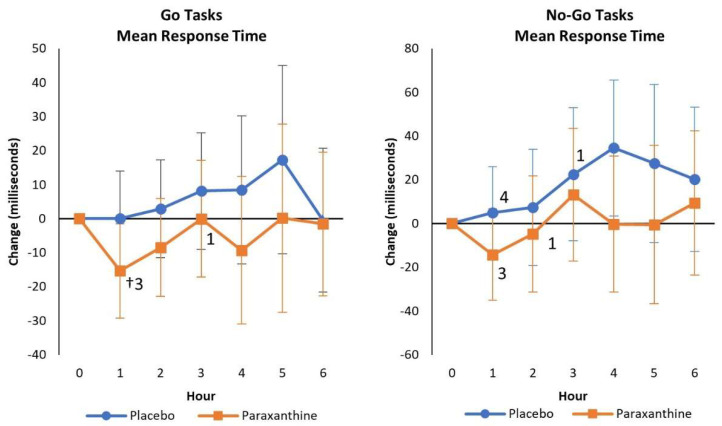
Mean changes with 95% confidence interval data from baseline in Go and No-Go mean response times. † represents *p* < 0.05 change from baseline values. Numbers 1, 2, 3, 4, 5, and 6 represent *p* < 0.05 differences from the hour values shown.

Table S3 presents Go Tasks and No-Go Tasks results that assess sustained attention, response control to visual stimuli, and impulsiveness. No significant overall or univariate treatment × time interaction effects were observed from GLM analysis using weight as a covariate, although some weak to moderate treatment x time effect sizes were observed. There was some evidence that PXN ingestion better maintained mean accuracy over time and Condition R Round 2 response time (e.g., PXN −25.1 [−52.2, 1.9] ms, *p* = 0.07 faster than PLA at 1 h) as well as Go Tasks mean response time (PLA 0.03 [−13.9, 13.9], *p* = 0.96; PXN −15.4 [−29.3, −1.5], *p* = 0.03 ms) after 1 h with PXN treatment, while Round 2 Condition *p* values and No-Go Tasks mean response time increased over time with PLA treatment (Figure 4).

#### 4.2.3. Sternberg Task Test

**Figure 5:**
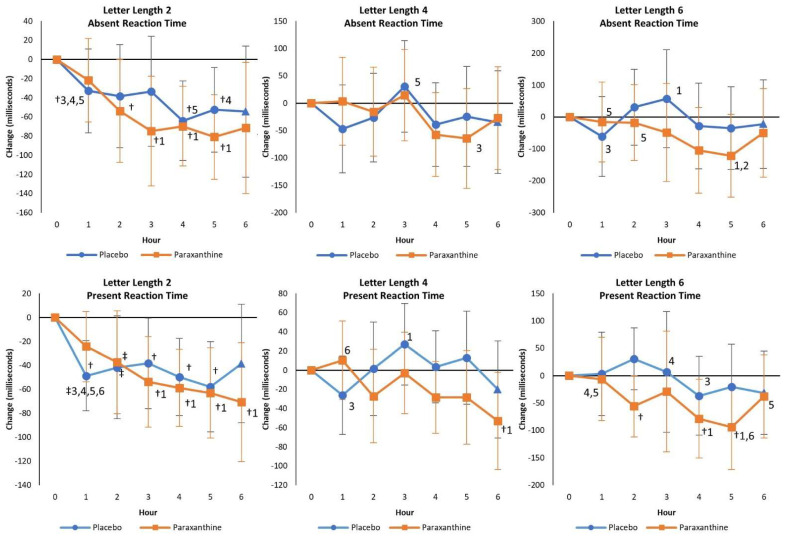
Mean changes with 95% confidence intervals in absent and present reaction times. † represents *p* < 0.05 change from baseline values. ‡ represents *p* > 0.05 to *p* < 0.10 change from baseline values. Numbers 1, 2, 3, 4, 5, and 6 represent *p* < 0.05 differences from the hour values shown.

Table S4 presents the Sternberg task test reaction time results. No significant overall or univariate treatment × time interaction effects were observed from GLM analysis using weight as a covariate in reaction time data. However, analysis of changes from baseline with 95% CIs (Figure 5) revealed that absent and present reaction times significantly decreased from baseline more consistently with PXN treatment, while response times generally did not change from baseline with PLA treatment. There was also evidence that PXN ingestion improved two-letter length absent and present reaction times over time as well as improving six-letter length absent reaction time after 2 h (PXN −86.5 ms [−165, −7.2], *p* = 0.03; PXN −9.0% [−18.1, 0.2], *p* = 0.05), suggesting that PXN enhanced the ability to store and retrieve random information of increasing complexity from short-term memory to a greater degree. Finally, a moderate treatment × time effect size (η_p_^2^ = 0.08) was observed in Trial 2 with reaction time, where PXN sustained vigilance during Trial 2 after 2 h (PXN 840 ms [103, 1576], *p* = 0.03) and 4 h (PXN 1466 ms [579, 2353], *p* = 0.002) compared to PLA. Moreover, as testing progressed, the response time improved during the 20 trials and over the course of the 6 h experiment in the PXN treatment, whereas it significantly increased in the PLA group, suggesting that PXN helped sustain attention (i.e., maintained reaction times, prevented mental fatigue).

#### 4.2.4. Psychomotor Vigilance Task Test

**Figure 6:**
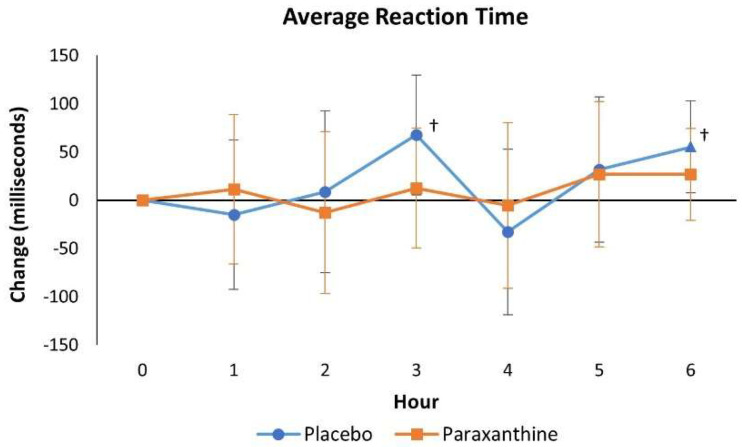
Mean changes with 95% confidence intervals in absent and present reaction times. † represents *p* < 0.05 change from baseline values.

Table S5 presents the psychomotor vigilance task test data. No significant overall or univariate treatment x time effects were observed. Analysis of mean changes from baseline with 95% CIs revealed that average reaction times with PLA treatment were increased from baseline after 3 and 6 h, while reaction times were maintained with PXN treatment (see Figure 6).

### 4.3. Safety Assessment

No subjective side effects or adverse events were reported by participants in response to the acute ingestion of PXN.

## 5. Discussion

Caffeine is a naturally occurring stimulant that is commonly consumed to promote wakefulness, alertness, and focus. However, there is individual variability in caffeine sensitivity and efficacy. Therefore, there has been interest in identifying alternatives to caffeine that may enhance cognition with less side effects. About 70% of CA is metabolized to PXN, and there is some evidence from animal studies that PXN may affect psychomotor and cognitive function. The purpose of this study was to examine whether acute ingestion of paraxanthine affects cognition, executive function, memory, and/or sustained attention in healthy human volunteers. If so, it may warrant additional dose effectiveness studies and comparative effectiveness studies to caffeine and/or other nootropic nutrients. This was accomplished by having participants undergo a battery of cognitive function tests that assessed different types of cognitive function prior to and following 1, 2, 3, 4, 5, and 6 h. The rationale was to determine which markers of cognitive function, memory, or attention may be affected and how long they might be impacted. We hypothesized that PXN would provide significant benefits on markers of cognitive function. The primary finding of this study was that a single 200 mg dose of PXN improved some markers of cognitive function and short-term memory and helped sustain attention, with no reported side effects. The following provides an additional discussion about the findings observed and areas for potential research.

Caffeine is one of the most commonly consumed ingredients throughout the world and is naturally found in coffee beans, tea leaves, cocoa beans, kola nuts, and guarana berries [38]. People of often consume caffeinated beverages and/or foods to help them wake up in the morning, delay fatigue during the day, and/or provide cognitive- or physiological-related performance benefits [39,40,41]. The pharmacokinetics and ergogenic value of caffeine on cognitive function and exercise performance has been well documented [6,14,40,42,43,44]. While most people tolerate caffeine well, some individuals are more sensitive to caffeine and/or experience unwanted side effects such as excessive jitteriness, nervousness, increased heart rate, palpitations, and/or increases in blood pressure [40,45,46]. This has raised concern about excessive consumption of caffeinated beverages and foods, particularly in more sensitive, younger, clinically vulnerable populations [47]. Research has suggested that individual variability in metabolizing caffeine may be related to the influence of the CYP1A2 gene on the enzyme responsible for caffeine metabolism [48,49]. In this regard, about 90% of caffeine is metabolized in the liver by P450 enzymes [50] which are coded by the CYP1A2 gene [48,49,50,51]. The clearance rate of caffeine is influenced by CYP1A2 activity [48,49,50,51]. Genetic polymorphisms (e.g., ADORA21, CYPIA2), smoking, liver disease, diet, pregnancy, the use of hormonal contraception in females, and alcohol intake have been reported to affect caffeine clearance and/or metabolism and thereby sensitivity to caffeine [20,48,52,53]. Consequently, there has been interest in identifying whether caffeine derivatives may have less impact on the central nervous system and serve as alternatives to caffeine in improving executive function, attention, and/or memory over time.

This study examined whether acute ingestion of 200 mg of PXN would affect cognition, memory, and/or vigilance. While two studies have been conducted in humans on the pharmacokinetics [6] and sympathomimetic properties [8] of PXN, we believe this is the first study to assess whether PXN ingestion affects cognition, memory, and/or vigilance. The first test administered was the Berg Wisconsin Card Sorting Test. This test assessed reaction time and accuracy in measuring thought, reasoning, learning, executive control, attention shifting, and impulsiveness [28,29]. The results reveal evidence that paraxanthine decreased the number of errors over time with differences noted from the placebo after 6 h of administration. These findings provide some evidence that paraxanthine helps sustain attention over time, thereby improving accuracy. The next test administered was the Go/No-Go test. This test evaluates the ability to sustain attention, control responses to visual stimulation, reaction time, and accuracy [26,27,30]. The results reveal that in comparison to a placebo, paraxanthine ingestion decreased the mean response time to Go Tasks after one hour of administration, while the response time increased over time with the placebo treatment. These findings provide additional evidence that paraxanthine ingestion may improve attention, reaction time, and accuracy. The Sternberg task test was then administered which assesses short-term/working memory involving cognitive control processes, reaction time, and accuracy [31,54,55,56]. This test assesses memory, reaction time, and accuracy of tasks requiring greater complexity by adding letter length challenges and performing 20 trials to assess attention and performance. Analysis of this cognitive challenge revealed that paraxanthine improved the reaction time from baseline more consistently than the placebo treatment, with reaction time improvements seen with shorter (two-letter) and longer (six-letter) challenges, which were consistent as the number of trials progressed. These findings provide some evidence that paraxanthine enhanced the ability to store and retrieve random information of increasing complexity from short-term memory to a greater degree, as well as helping to sustain attention (i.e., maintained reaction times, prevented mental fatigue). Finally, participants performed the psychomotor vigilance task test which assesses sustained attention reaction times through responses to visual stimuli [32,33,34]. The results reveal that the average reaction time was better maintained over time with paraxanthine. The benefit appeared to peak in about 3–5 h. No side effects were reported, suggesting that acute administration of paraxanthine was well tolerated.

## 6. Conclusions

The present findings support contentions that paraxanthine may influence memory, cognition, and attention and therefore have nootropic properties. The strength of this study is that it evaluated whether the primary metabolite of caffeine affects cognition, memory, or attention. This may increase our understanding about how caffeine and its metabolites influence cognitive and executive function. The limitation of this study is that we only assessed the impact of one oral dose (200 mg) of PXN on primary and secondary outcome measures. Additional research will need to examine minimal effective and optimal doses and the impact of longer periods of supplementation and compare paraxanthine to caffeine to determine if it can serve as an effective nootropic alternative with less side effects. The results of this study are promising and warrant additional research.

## Data Availability

Data and/or statistical analyses are available upon request on a case-by-case basis for non-commercial scientific inquiry and/or educational use as long as IRB restrictions and research agreement terms are not violated.

## Supplementary Materials

The following are available online at https://www.mdpi.com/article/10.3390/nu13113980/s1, Table S1: Participant demographic data, Table S2: Berg Wisconsin Card Sorting Test results, Table S3: Go/No-Go task test results, Table S4: Sternberg task test reaction time results, Table S5: Psychomotor vigilance task test results.
